# Polyamine-Based Nanostructures Share Polyamine Transport Mechanisms with Native Polyamines and Their Analogues: Significance for Polyamine-Targeted Therapy

**DOI:** 10.3390/medsci10030044

**Published:** 2022-08-22

**Authors:** Cassandra E. Holbert, Jackson R. Foley, Ao Yu, Tracy Murray Stewart, Otto Phanstiel, David Oupicky, Robert A. Casero

**Affiliations:** 1 Sidney Kimmel Comprehensive Cancer Center, Baltimore, MD 21231, USA; 2 Drug Delivery and Nanomedicine, Department of Pharmaceutical Sciences, University of Nebraska Cancer Center, Omaha, NE 68105, USA; 3 Department of Medical Education, University of Central Florida, Orlando, FL 32827, USA

**Keywords:** polyamine, polyamine analogue, drug transport, cancer therapy, nanoparticle, polyamine transport, drug delivery system, nanopolyamine

## Abstract

Polyamines are small polycationic alkylamines involved in many fundamental cellular processes, including cell proliferation, survival, and protection from oxidative stress. Polyamine homeostasis is tightly regulated through coordinated biosynthesis, catabolism, and transport. Due to their continual proliferation, cancer cells maintain elevated intracellular polyamine pools. Both polyamine metabolism and transport are commonly dysregulated in cancer, and as such, polyamine analogues are a promising strategy for exploiting the increased polyamine requirement of cancer cells. One potential polyamine analogue resistance mechanism is the downregulation of the poorly defined polyamine transport system. Recent advances in nanomedicine have produced nanostructures with polyamine analogue-based backbones (nanopolyamines). Similar nanostructures with non-polyamine backbones have been shown to be transported by endocytosis. As these polyamine-based nanoparticles could be a method for polyamine analogue delivery that bypasses polyamine transport, we designed the current studies to determine the efficacy of polyamine-based nanoparticles in cells lacking intact polyamine transport. Utilizing polyamine transport-deficient derivatives of lung adenocarcinoma lines, we demonstrated that cells unable to transport natural polyamines were also resistant to nanopolyamine-induced cytotoxicity. This resistance was a result of transport-deficient cells being incapable of importing and accumulating nanopolyamines. Pharmacological modulation of polyamine transport confirmed these results in polyamine transport competent cells. These studies provide additional insight into the polyamine transport pathway and suggest that receptor-mediated endocytosis is a likely mechanism of transport for higher-order polyamines, polyamine analogues and the nanopolyamines.

## 1. Introduction

Polyamines are small, polycationic alkylamines involved in numerous critical cell processes including proliferation, cell survival, protection from oxidative stress, and nucleic acid synthesis and stabilization [1,2]. In cancer, the metabolism of and requirement for polyamines are frequently dysregulated [3]. As polyamines have the ability to simultaneously affect multiple fundamental processes, the intracellular concentrations of naturally occurring polyamines are normally tightly regulated through coordinated biosynthesis, catabolism, and transport. While the polyamine biosynthesis and catabolism pathways have been studied in depth, the mammalian polyamine transport system remains poorly defined [4,5,6].

The naturally occurring polyamines, spermidine (SPD), spermine (SPM) and their diamine precursor putrescine have a net positive charge at physiological pH and are known to interact with free radicals and many negatively charged macromolecules including certain proteins and nucleic acids [7]. This protonation prevents polyamines from undergoing efficient passive diffusion through cell membranes. Mammalian polyamine transport is an energy-dependent process that has been biochemically categorized; however, little is known about the molecular players involved in transport. While extensive progress has been made in the molecular characterization of the transport system in prokaryotes, yeast and trypanosomatids, this success has yet to fully transfer to the metazoan sphere.

Currently, there are three putative molecular models for the polyamine transport system, all of which, in part, correspond to the available biochemical data. Polyamine transport in yeast and prokaryotes relies on various plasma membrane polyamine permeases (PMPPs). The most well-defined model of metazoan polyamine transport suggests a two-step process beginning with surface transport by an undefined PMPP followed by rapid sequestration into polyamine sequestering vesicles (PSVs) [6]. Sequestration into these acidic PSVs (identical to the late endosomes and lysosomes of endocytosis) utilizes a proton:polyamine exchange through vesicular H^+^-coupled polyamine antiporters [6]. A secondary model, specific only to spermine, suggests that glypican-1 acts as a high-affinity spermine receptor [4]. Glypican-1 activates receptor-mediated endocytosis, which internalizes and sequesters spermine into intracellular PSVs from which it can be subsequently freed through nitric oxide-mediated oxidation [4]. The most recent model was proposed in gastrointestinal tissues [8]. In this model, putrescine uptake is mediated by caveolar endocytosis and nitric oxide production, and the solute carrier protein SLC3A2 is implicated in both import and export of putrescine [5,8,9]. Recent advances have identified additional proteins involved in polyamine transport, including ATP13A2 as a lysosomal polyamine exporter and ATP13A3 in the import of spermidine and spermine in pancreatic cancers [10,11,12]. It is hypothesized that eukaryotic cells do not utilize a singular molecular mechanism for polyamine transport, and it is possible that all three of these proposed models are employed in some form.

Dysregulation of polyamine homeostasis is implicated in a variety of disease states including neurodegenerative, infectious, and cancerous [13,14,15]. Because polyamines are absolutely required for proliferation and many oncogene-driven cancers rely on an elevated intracellular polyamine pool, both polyamine metabolism and transport are frequently dysregulated in cancer [16]. Many cancer types show a correlation between increased cell proliferation and an increase in intracellular polyamines through upregulation of polyamine biosynthesis and transport [3,17,18]. This dependence on increased polyamine concentration makes polyamine metabolism an appealing target for cancer therapeutics. Using polyamine analogues to exploit the tight self-regulation of polyamine homeostasis is a promising strategy for therapeutic benefit in neoplastic conditions. Polyamine analogues can be divided into numerous classes with a main class involving the alkylation of the primary amine groups of spermine. These compounds are either symmetrically substituted bis (ethyl) analogues or unsymmetrically substituted and compete with endogenous polyamines for uptake and, upon intracellular accumulation, stimulate the catabolism of higher order polyamines and reduce polyamine biosynthesis [19]. The cytotoxicity associated with these analogues is partially attributable to significant induction of polyamine catabolism through tumor type-specific induction of spermidine/spermine N^1^-acetyltransferase (SSAT) and spermine oxidase (SMOX) activity [16]. Increased SSAT activity rapidly reduces natural spermidine and spermine content in susceptible cells, and the byproducts of SMOX activity include the reactive oxygen species precursor hydrogen peroxide and the highly toxic aldehyde 3-aminopropanal. Accumulation of these byproducts leads to ROS-induced apoptosis [16].

**Figure 1:**
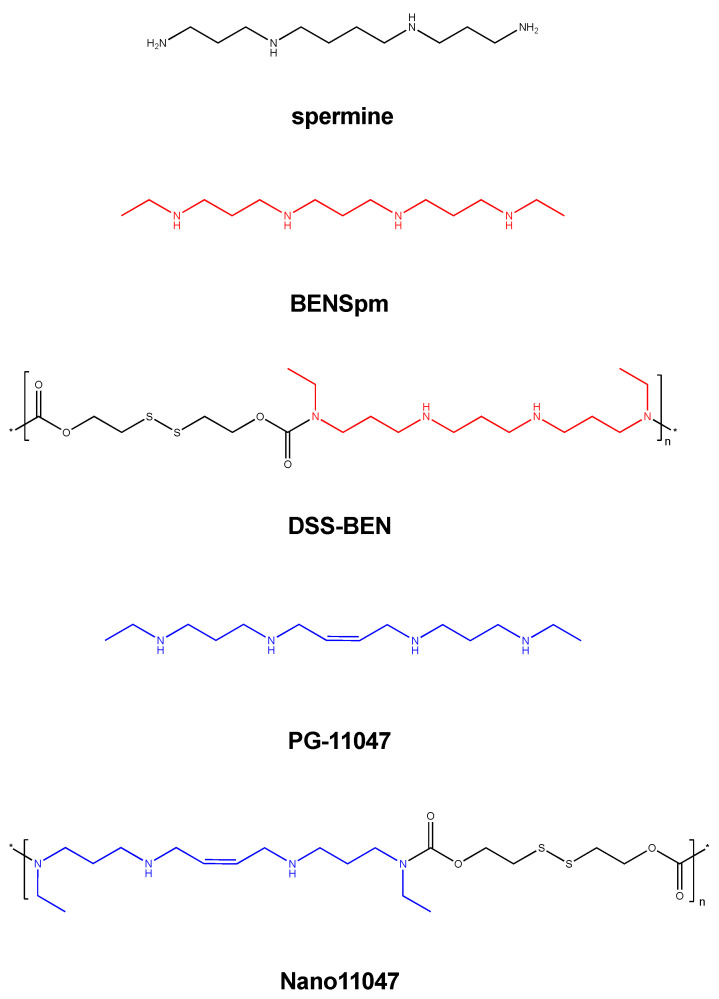
**Structures of polyamine analogues and nanoparticle derivatives.** Both bis(ethyl)norspermine (BENSpm) and PG-11047 are spermine-derived polyamine analogues. DSS-BEN is a nanoparticle compound synthesized from BENSpm, and Nano11047 is derived from PG-11047. Both nanoparticles undergo intracellular thiolytic reduction, subsequently disassembling and releasing the parent compound, BENSpm or PG-11047, respectively.

Several symmetrically substituted polyamine analogues exhibit polyamine metabolism modulation in vitro and are efficacious against various cancers both preclinically and clinically. These compounds include diethyl dihydroxyhomospermine (SBP-101), N^1^,N^12^-bis(ethyl)-cis-dehydrospermine (PG-11047) and N^1^,N^11^-bis(ethyl)norspermine (BENSpm) [20,21,22,23,24]. SBP-101 is known to influence polyamine metabolism in vitro and in vivo, and is currently being evaluated for efficacy against pancreatic cancer in a phase 2/3 clinical trial [24,25,26,27]. PG-11047 is a second-generation analogue containing a central *cis* double bond conformational restriction aimed at reducing off-target effects seen in clinical BENSpm trials [20,21,22,28,29]. Preclinical animal models have shown PG-11047 to delay tumor progression and extend survival; however, the clinical trials (with potentially suboptimal dosing schedules) to date have only managed to maintain stable disease [30,31]. Renewed interest in clinically efficacious polyamine analogues has led to the production of PG-11047 and BENSpm nanoparticles (Figure 1).

Single-agent cancer therapeutics are often of limited success due to complexity stemming from tumor heterogeneity and acquired drug resistance. Clinical focus in the polyamine field has shifted from single agents to combination therapies in an attempt to utilize more targeted drug delivery as well as produce longer lasting clinical responses [32,33,34,35]. Recent advances in polymeric nanoparticles have produced polyamine-based self-immolative polycations capable of affecting polyamine metabolism while simultaneously acting as a delivery system for therapeutic nucleic acids [33,36,37,38]. Two of these nanopolyamines, Nano11047 and DSS-BEN, are synthesized from PG-11047 and BENSpm, respectively. These dendritically branched nanopolyamines are similar to other characterized cationic dendrimers and were previously reported to be transported by endocytosis followed by endosomal escape and self-immolation to their parent compounds [36,39]. A common mechanism of resistance to polyamine analogues in cancer cells is the downregulation of polyamine transport. As these nanopolyamines were anticipated to be accumulated through general endocytosis as opposed to selective polyamine transport, they were initially thought to provide a potential method of polyamine analogue delivery into cells that have downregulated or lost polyamine transport. We designed the current studies to determine the efficacy of nanopolyamines in cells lacking intact polyamine transport and to investigate the potential for overlap with the current models of polyamine transport. Surprisingly, our results indicate that the nanopolyamines are transported by the same transport system as the natural polyamines and their monomer analogues.

## 2. Materials and Methods

### 2.1. Cell Lines and Culture Conditions

Wildtype A549 and H157 lung adenocarcinoma cells were purchased from ATCC (Manassas, VA, USA) and were maintained in RPMI 1640 containing 10% bovine calf serum (Gemini Bio-Products, Sacramento, CA, USA) and penicillin/streptomycin at 5% CO_2_ and 37 °C. A549 and H157 transport-deficient cells (A549R and H157R) and the corresponding controls (A549G and H157G) were previously developed in our lab [40] and maintained as listed above with the addition of 250 μg/mL G418, an aminoglycoside antibiotic.

### 2.2. Cell Viability Assays

A549 or H157 cells were seeded in triplicate wells per condition of a 96-well plate at a density of 3 × 10^4^ cells/well and allowed to attach overnight. Cells were then treated with 100 μL of fresh medium containing the appropriate concentration of PG-11047, Nano11047, BENSpm, or DSS-BEN. Following the indicated incubation times, cell viability was determined using the CellTiter-Blue Cell Viability Assay (Promega, Madison, WI, USA). Fluorescence was measured using a SpectraMax M5 (Molecular Devices, San Jose, CA, USA). IC_50_ values for all treated cell lines were calculated by GraphPad Prism (v. 8.3.1) (GraphPad Software, La Jolla, CA, USA) using nonlinear regression for a normalized response.

### 2.3. Polyamine-Based Nanoparticle Synthesis

The synthesis of DSS-BEN and Nano11047 was performed as previously described [37]. Briefly, bis(2-hydroxyethyl) was dissolved in a mixture of dichloromethane (DCM) and tetrahydrofurane on ice. Next, a solution of 1,1′-carbonyldiimidazole in DCM was added followed by a 1-h incubation on ice. BENSpm (or PG-11047) in DCM was added and the reaction was allowed to proceed at 45 °C for 18 h. After cooling to room temperature, the final product was precipitated in 25 mL of diethyl ether, dried under vacuum and dispersed in 0.1 mM HCl, followed by extensive dialysis against 0.1 mM HCl and then pure water before lyophilization. The product was characterized by ^1^H-NMR and size-exclusion chromatography. The molecular weights, as determined by dynamic light scattering, of DSS-BEN and Nano11047 are 3.8 kDa and 7.2 kDa, respectively [37].

### 2.4. Analysis of Intracellular Native Polyamine Concentrations, Polyamine Analogue Concentrations and Catabolic Polyamine Enzyme Activity

Treated cells were lysed and intracellular polyamine (putrescine, spermidine and spermine) or polyamine analogue (PG-11047 and BENSpm) concentrations were determined by reverse-phase high performance liquid chromatography (HPLC) following acid extraction and pre-column dansylation of the lysate as previously described by Kabra et al. [41]. SSAT activity was determined as previously described [24].

### 2.5. DFMO Growth Curves and Nanopolyamine Co-Treatment

To confirm that A549R cells are polyamine transport deficient, treatment with 5 mM DFMO with and without 2 μM spermidine supplementation was completed over 96 h. All cells were treated with 1 mM aminoguanidine to inhibit oxidation of spermidine by serum amine oxidase [42]. Cells were trypsinized and viable cells were counted with an automated cell counter every 24 h for 96 h. Cell viability was determined by the ability of cells to exclude trypan blue.

A549 cells were seeded at a density of 4 × 10^5^ cells/T75 and allowed to attach overnight. The media was then replaced with RPMI supplemented with 5 mM DFMO (provided by Professor Patrick M. Woster, Medical University of South Carolina). Following 72 h of DFMO pre-treatment, Nano11047 (20 ng/mL) was added directly to the media (freshly replenished with 5 mM DFMO). Following an additional 48 h of incubation time, cells were lysed and intracellular polyamine analogue concentrations were determined as described above.

### 2.6. Polyamine Transport Inhibition

The polyamine transport inhibitor, trimer44NMe was synthesized as previously reported [43]. A549 cells were co-treated with 75 μM trimer44NMe and either PG-11047 (10 μM), Nano11047 (20 ng/mL), BENSpm (10 μM) or DSS-BEN (10 ng/mL). Following 48 h of incubation, cells were lysed and intracellular polyamine analogue concentrations were determined as described above.

### 2.7. Statistical Analysis

Statistical analysis was completed with GraphPad Prism (v. 8.3.1) (GraphPad Software, La Jolla, CA, USA). All data sets passed the Shapiro-Wilk test for normality. Cell viability was analyzed using the Holm-Sidak method of multiple unpaired *t*-tests and intracellular polyamine analogue concentrations were analyzed using a Welch’s unpaired *t*-test. *p*-value indications are as follows: * *p* < 0.05; ** *p* < 0.01; *** *p* < 0.001; **** *p* < 0.0001.

## 3. Results

### 3.1. Polyamine Transport-Deficient Cells Are Resistant to Nanopolyamine and Polyamine Analogue-Induced Cytotoxicity

Nanopolyamines were synthesized utilizing spermine analogues linked by a self-immolative BHED linker [36]. This disulfide linker is readily cleaved in the reductive intracellular environment and yields unmodified BENSpm or PG-11047 [36,37]. DSS-BEN is synthesized from bis(ethyl)norspermine (BENSpm), and Nano11047 is derived from PG-11047, a less-toxic, second-generation spermine analogue (Figure 1). It was previously reported that treatment with DSS-BEN induces polyamine catabolism and has anti-cancer activity both in vitro and in vivo [37]. Nano11047 had similar antitumor effects on polyamine metabolism in lung cancer cell lines.

**Figure 2:**
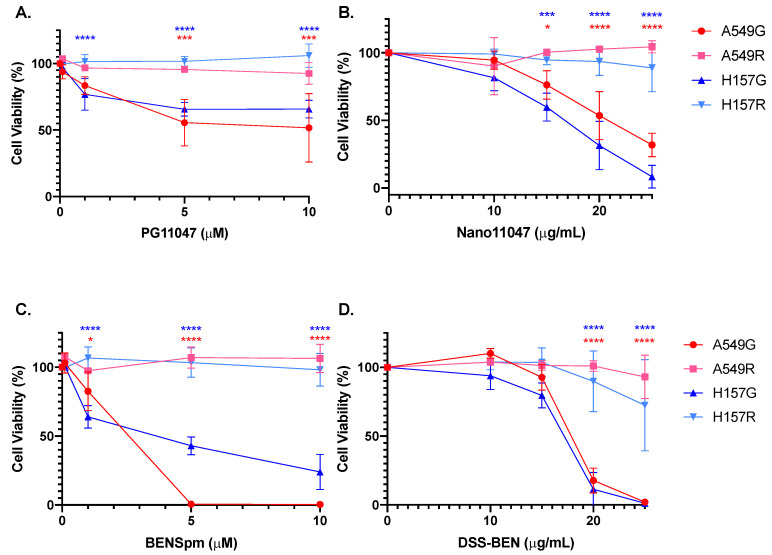
**Polyamine transport deficient cells evade nanopolyamine cytotoxicity.** Polyamine transport-competent (G) and polyamine transport-deficient (R) cell lines were treated for 96-h with increasing concentrations of nanopolyamines or their parental polyamine analogue. Only cells capable of transporting polyamines exhibited cytotoxicity following treatment with PG-11047 (**A**) and BENSpm (**C**). Similarly, nanopolyamines Nano11047 (**B**) and DSS-BEN (**D**) reduced cellular viability only in cell lines with intact polyamine transport. Red asterisks indicate results of t-tests between A549G and A549R, while blue asterisks indicate results of t-tests between H157G and H157R. *p*-value indications are as follows: * *p* < 0.05; *** *p* < 0.001; **** *p* < 0.0001.

Transport-competent and transport-deficient derivatives of two human lung adenocarcinoma lines, A549 and H157, were used throughout the current studies [40]. The polyamine transport-deficient lines (A549R and H157R) as well as transport-competent, G418-resistant controls (A549G and H157G) were used to determine the cytotoxicity of Nano11047 and DSS-BEN. Cells were treated for 96 h with concentrations ranging from 1 μM to 10 μM of the parental polyamine analogue (PG-11047 or BENSpm) or 10 to 25 μg/mL of Nano11047 or DSS-BEN. IC_50_ values were calculated for each treatment (Table 1). PG-11047 caused an approximately 50% reduction in cell viability following 5 μM of treatment in both A549G and H157G cells (Figure 2A), while the transport-deficient lines (A549R and H157R) showed no reduction in cell viability with up to 10 μM of PG-11047 treatment. Cell viability was significantly reduced to near 50% following 20 μg/mL of Nano11047 in A549G or 15 μg/mL of Nano11047 in H157G (Figure 2B). Nano11047 treatment did not result in decreased cell viability in either A549R or H157R cell lines.

Similarly, treatment with 5 μM of BENSpm was sufficient to reduce H157G cell viability to 50% and eliminate A549G viability (Figure 2C), while BENSpm treatment had no effect on transport-deficient cell lines up to 10 μM. DSS-BEN treatment markedly reduced viability (80%) in transport-competent cell lines, whereas treatment caused only a limited decrease in viability in transport-deficient cell lines (Figure 2D). These results indicate that these two nanopolyamines are not cytotoxic to cells incapable of transporting polyamines.

### 3.2. Polyamine Transport-Deficient Cells Are Unable to Accumulate Nanopolyamines or Their Parent Compounds

**Figure 3:**
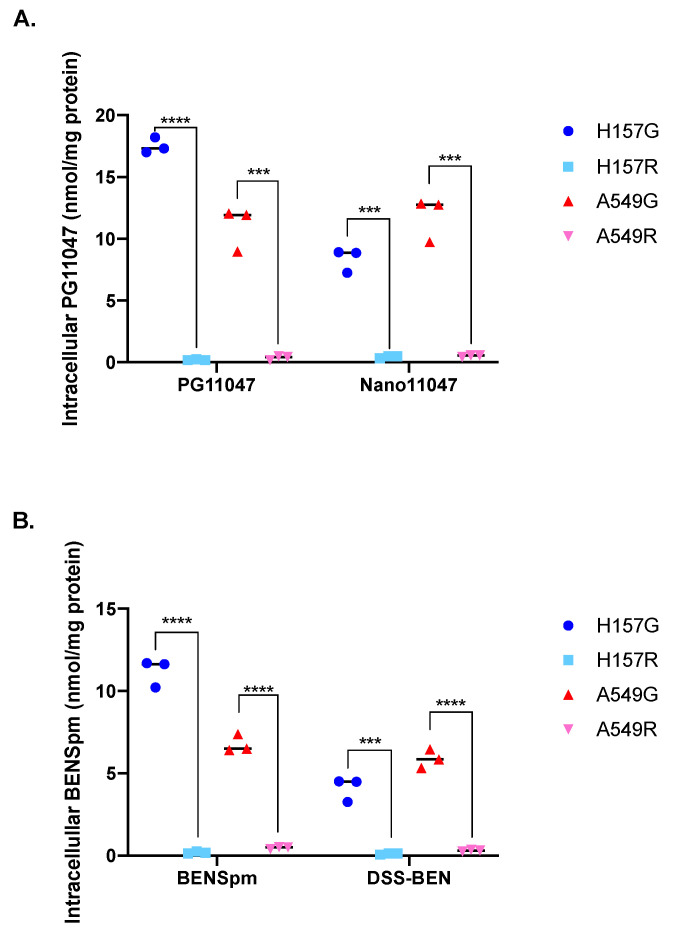
**The parental analogues of nanopolyamines do not accumulate in cells deficient in polyamine transport.** Following 48-h treatment with either a nanopolyamine or the parental polyamine analogue, intracellular concentrations of the parental compounds were measured by via N-dansylation and high-performance liquid chromatography (HPLC). PG-11047 is detectable intracellularly following treatment with either PG-11047 or Nano11047 (**A**) in transport-competent cells (A549G/H157G). Transport-deficient cells (H157R/A549R) do not accumulate PG-11047 following either PG-11047 or Nano11047 treatment (**A**). Similarly, only transport-competent cells accumulated BENSpm following treatment with either BENSpm or DSS-BEN (**B**). *p*-value indications are as follows: *** *p* < 0.001; **** *p* < 0.0001.

Once accumulated by transport-competent cells, the nanopolyamines immediately undergo intracellular thiolytic reduction by GSH, subsequently disassembling and releasing their respective parent compound [37]. The intracellular concentrations of these parent compounds, PG-11047 and BENSpm, can then be quantified by high performance liquid chromatography (HPLC). Cells were treated for 72 h with either 10 μM of PG-11047 or BENSpm, 20 μg/mL of Nano11047, or 10 μg/mL of DSS-BEN and collected for HPLC analysis. Following PG-11047 and Nano11047 treatment, H157G cells had accumulated intracellular PG-11047 concentrations of 18 nmol/mg protein and 8 nmol/mg total protein, respectively (Figure 3A). PG-11047 and Nano11047 treatment in A549G cells yielded an average concentration of 12 nmol/mg protein following either treatment. Neither polyamine-transport deficient cell line accumulated intracellular PG-11047 following treatment with either the parental compound or Nano11047 (Figure 3A). The intracellular concentration of BENSpm in H157G cells following treatment was 11 nmol/mg, while DSS-BEN treatment resulted in an accumulation of approximately 4.5 nmol/mg BENSpm (Figure 3B). Treatment with either BENSpm or DSS-BEN yielded an intracellular accumulation of 6 nmol/mg BENSpm in transport-competent A549 cells. Similar to results with PG-11047 and Nano11047, no intracellular BENSpm accumulation was detected in polyamine-transport-deficient cells treated with either BENSpm or DSS-BEN (Figure 3B).

**Figure 4:**
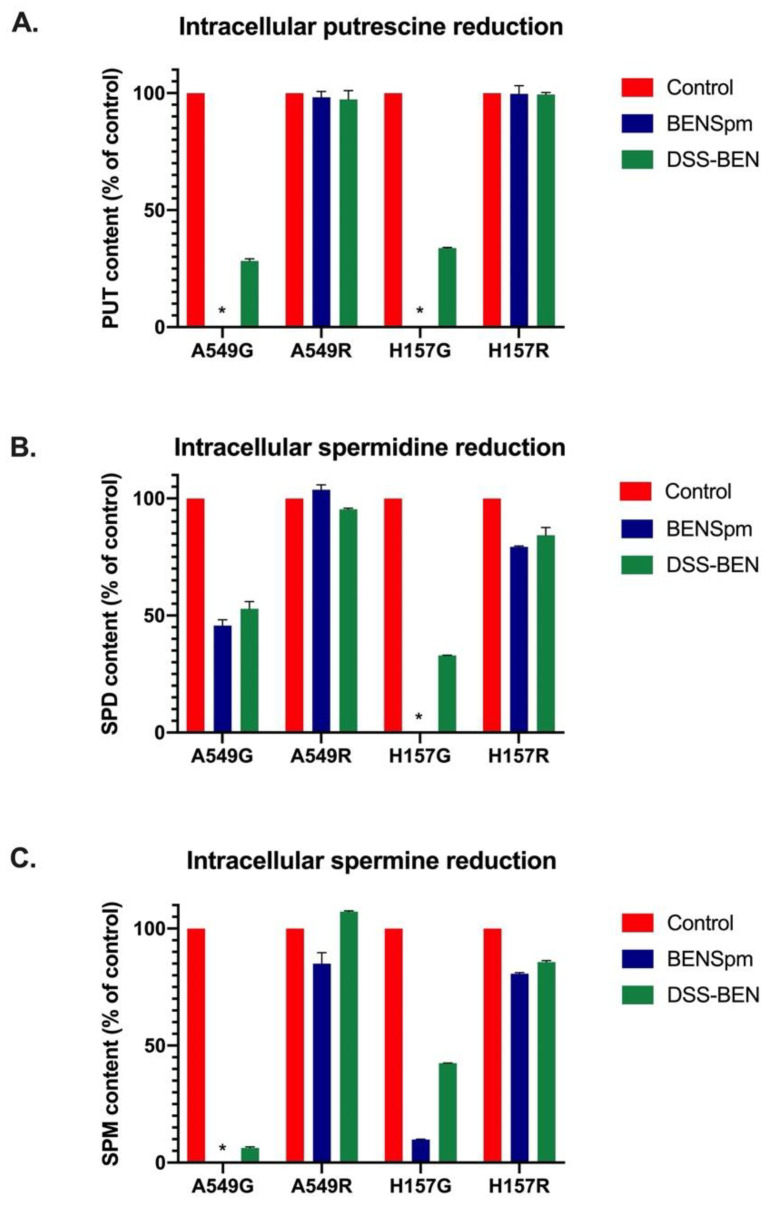
**Intracellular polyamine content is reduced by polyamine analogues and nanopolyamines only in the presence of intact polyamine transport.** Following 48-h treatment with either polyamine analogue BENSpm (10 μM) or its nanopolyamine DSS-BEN (10 μg/mL), cells were lysed and intracellular polyamine content was determined by HPLC. Treatment with BENSpm in both transport-competent cell lines, A549G and H157G, produced a complete reduction in putrescine to below detectable levels (**A**). DSS-BEN treatment also resulted in decreased putrescine levels in these cells, but neither treatment reduced putrescine levels in transport-deficient cell lines A549R and H157R. Similarly, BENSpm and DSS-BEN treatment reduced spermidine (**B**) and spermine levels (**C**) in transport-competent cell lines but had no effect on transport-deficient cell lines. * indicates no detection/below level of detection.

Upon intracellular accumulation, polyamine analogues influence polyamine metabolism and lead to an overall decrease in intracellular polyamine levels [16]. Following 24 h of BENSpm or DSS-BEN treatment, cells were lysed, and intracellular polyamine content was determined. A549G and H157G cells showed a complete reduction in putrescine to below detectable levels following BENSpm treatment and more than 70% reduction in putrescine content following DSS-BEN nanopolyamine treatment (Figure 4A). Neither transport-deficient cell line exhibited any reduction in putrescine content following treatment with either BENSpm or DSS-BEN. Spermidine levels were decreased by approximately 50% following BENSpm or DSS-BEN treatment in A549G cells (Figure 4B). Their transport-deficient counterpart, A549R, displayed no change in intracellular spermidine content. Similarly, treatment with BENSpm or DSS-BEN decreased spermidine levels dramatically in H157G cells but produced little effect in H157R cells. Spermine levels were reduced in A549G and H157G cells following treatment with either DSS-BEN or its parent analogue, BENSpm, while A549R and H157R cells exhibited no intracellular spermine reduction (Figure 4C).

### 3.3. Effects of Polyamine Transport System Modulators on Uptake of Nanopolyamines

Polyamine transport can be biochemically altered using a variety of compounds. While the direct target of polyamine transport inhibitors is unknown, they are still valuable tools for studying polyamine uptake. Trimer44NMe is a polyamine transport inhibitor known to block the uptake of the natural polyamines in mammalian cells [43]. Trimer44NMe alone shows limited toxicity in vitro but inhibits the uptake of exogenously supplemented putrescine, SPD, and SPM [43]. α-Difluoromethylornithine (DFMO) is an irreversible inhibitor of ornithine decarboxylase (ODC), one of the rate-limiting steps of polyamine biosynthesis [44,45,46,47]. The inhibition of ODC by DFMO triggers a compensatory response that upregulates the transport of exogenous polyamines. This has limited the success of DFMO as a monotherapy, but trimer44NMe and DFMO have shown success in vitro and in vivo as a combination approach to polyamine-based cancer therapy [43]. Both inhibitors provide opportunities to further investigate the role of polyamine transport in polyamine-based nanoparticle uptake.

**Figure 5:**
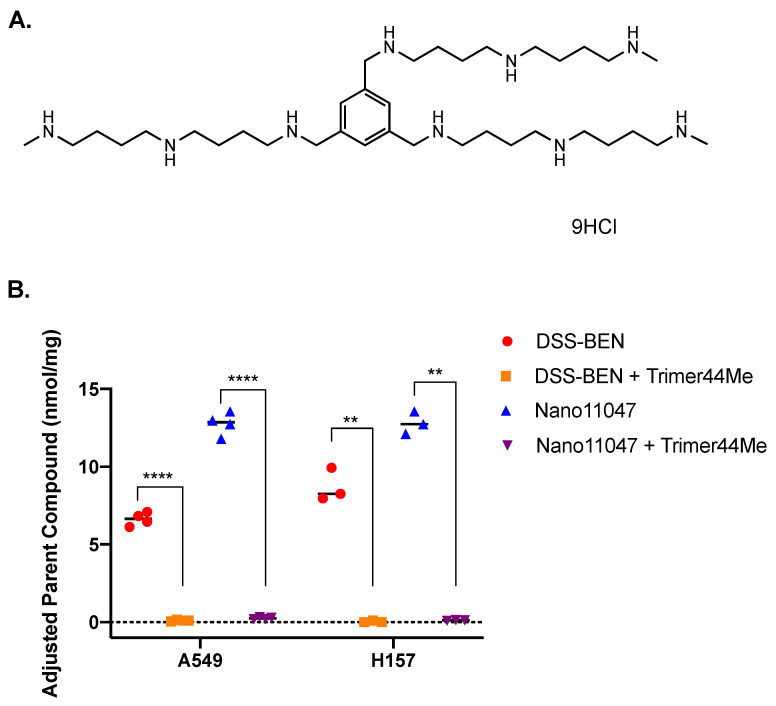
**Pharmacological inhibition of polyamine transport is sufficient to block analogue accumulation following nanopolyamine treatment.** Lung adenocarcinoma cell lines, H157 and A549, were co-treated for 48 h with trimer44NMe, a polyamine-transport inhibitor (**A**), and either DSS-BEN or Nano11047. The intracellular concentration of the parent compound (BENSpm or PG-11047, respectively) was measured by HPLC following treatment. Parent compound accumulated in wildtype H157 and A549 cells following DSS-BEN or Nano11047 addition, but co-treatment with trimer44NMe (75 μM) blocked this accumulation (**B**). *p*-value indications are as follows: ** *p* < 0.01; **** *p* < 0.0001.

We used wildtype cells with intact polyamine transport systems to determine the effects of trimer44NMe or DFMO on uptake of nanopolyamines. Cotreatment of wildtype A549 cells with trimer44NMe and DSS-BEN reduced the intracellular concentration of BENSpm from 6.3 nmol/mg protein to 0.12 nmol/mg protein (Figure 5). Cotreatment of H157 cells significantly reduced the intracellular concentration of BENSpm from 9.1 nmol/mg protein to below the detectable limit (Figure 5). Cotreatment of trimer44NMe and Nano11047 exhibited a similar trend. Cotreatment decreased intracellular PG-11047 levels from 12.4 to 0.29 nmol/mg protein in A549 cells and from 13.15 to 0.13 nmol/mg protein in H157 cells.

**Figure 6:**
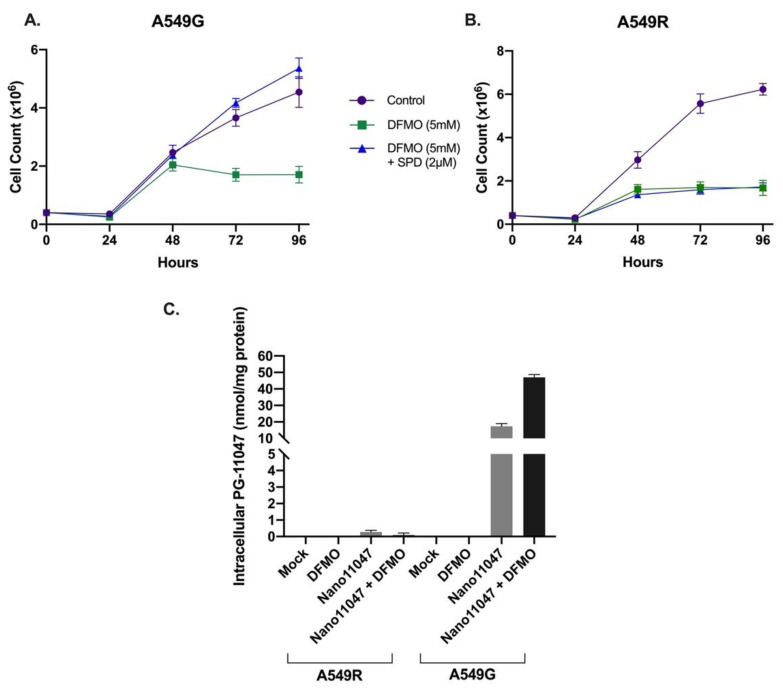
**DFMO does not upregulate nanopolyamine uptake in transport-deficient cells nor can DFMO-induced cyto-stasis be rescued by exogenous polyamines in transport-deficient cells.** Cells were treated with 5 mM DFMO +/− 2 μM spermidine and counted every 24 h for 96 h. All cells were treated with 1 mM aminoguanidine to inhibit serum amine oxidase. DFMO treatment slowed the growth of transport competent A549G cells, but normal growth was restored with the addition of exogenous spermidine (**A**). The growth of DFMO-treatedA549R transport-resistant cells was not rescued with spermidine supplementation (**B**). Following 72 h of DFMO pretreatment, A549 cells were treated for 48 h with Nano11047. Cells were lysed and intracellular PG-11047 content was determined (**C**). A549R cells showed no accumulation of the parent compound, PG-11047, regardless of DFMO treatment. A549G cells accumulated approximately 15 nmol/mg PG-11047 when treated with Nano11047 alone. Intracellular PG-11047 accumulation increased 3-fold when cells were pre-treated with DFMO to upregulate transport.

DFMO is known to induce cytostasis in some tumors and treatment of cells in vitro reduces cellular growth rate [48,49]. Normal growth, however, can be achieved with exogenous polyamine supplementation [50,51]. Spermidine supplementation of transport-competent A549G lung adenocarcinoma cells was sufficient to rescue DFMO-induced growth inhibition (Figure 6), but the A549R transport-deficient cell line did not exhibit growth rescue following spermidine supplementation. Consistent with its known ability to upregulate polyamine transport, DFMO pre-treatment upregulated Nano11047 uptake three-fold in A549G; however treatment of transport-deficient A549R cells with DFMO had no effect on the uptake of Nano11047 (Figure 6).

## 4. Discussion

Cancer cells depend on aberrant polyamine metabolism to maintain the increased intracellular polyamine requirement necessary for continuous proliferation [16]. Many cancers increase intracellular pools through upregulation of the polyamine biosynthetic pathway and/or upregulation of polyamine import [16]. Attempts have been made to inhibit biosynthesis enzymes, most notably the inhibition of ODC by DFMO; however, the difficulties associated with monotherapeutic enzyme inhibition have led to expanded interest in compounds that further regulate polyamine metabolism. Polyamine analogues are recognized and transported by the polyamine transport system but are dissimilar enough in structure that they are unable to functionally replace the natural polyamines resulting in cytotoxicity [13]. Numerous polyamine analogues have been developed, including PG-11047 and BENSpm, that inhibit tumor growth and exhibit cytotoxicity in preclinical models. Unfortunately, with the dosing schedules used to date, the best clinical outcome with either drug as a monotherapy has been stable disease in a limited number of patients [13,21,30].

The current study describes the requirement of intact polyamine transport in order to uptake the newly synthesized nanopolyamines, Nano11047 and DSS-BEN. It was previously shown that the transport of other polycationic-based nanoparticles occurs through generalized endocytosis. Therefore, to evaluate the efficacy of the nanopolyamines as a drug delivery system in polyamine transport-deficient cells, we designed the current studies. Utilizing lung adenocarcinoma cells with polyamine transport deficiencies, we determined that the nanopolyamines, similarly to their associated parental analogue compounds, only exhibited cytotoxic effects on cells with intact polyamine transport machinery. This was directly correlated with the uptake and subsequent disassembly of the nanopolyamines as determined by HPLC analysis. Significant intracellular analogue accumulation occurred in cells capable of polyamine transport, while the transport-deficient cell lines had poor accumulation of the analogues following nanopolyamine treatment. Polyamine transport-competent cells accumulated both parent analogues and their nanopolyamine counterparts, resulting in reduction of intracellular polyamine levels. Transport-resistant cell lines were impervious to the influence of polyamine analogues and nanopolyamines on polyamine pools because the cells were unable to uptake the compounds from the media.

A limitation to the transport-deficient lung adenocarcinoma cell line models is that the underlying molecular mechanism for their transport deficiency is not known [40]. However, we confirmed the results obtained in the transport-deficient cells using the alternative method of pharmacologically inhibiting polyamine transport as well as pharmacologically upregulating transport through DFMO. Uptake of nanopolyamines and the accumulation of their parental analogue was completely prevented in cells treated with trimer44NMe, a polyamine transport inhibitor, thus confirming that the nanopolyamines use the same transport system as do their parent monomer analogues and the natural polyamines. Similarly, DFMO, which is known to stimulate polyamine import, enhanced uptake of the nanopolyamines in wild type, transport competent cells.

While various substituted polyamines are known to utilize polyamine transport, it was anticipated that, due to their size and the behavior of similar nanoparticles, polyamine-based polycationic nanoparticles would bypass polyamine transport machinery and be accumulated by generalized endocytosis [36,39,52,53]. This study demonstrates, however, that the nanopolyamines utilize the same polyamine transport machinery for entry into mammalian cells as do the native polyamines and polyamine analogues. The data associated with polycationic nanoparticles similar to Nano11047 and DSS-BEN, as well as the size of the nanopolyamines, suggest that a form of endocytosis is the most likely mechanism of cellular uptake of these nanopolyamines [39,54,55,56,57]. If this hypothesis proves to be true, the data from these studies indicate that the natural polyamines are also taken up by a form of endocytosis, supporting the models of metazoan polyamine transport that involve endocytosis as the method of cell entry [4,8]. The glypican-1 model described previously suggests endocytosis followed by PSV sequestration. This model is specific for spermine, making it highly relevant for our purposes since both of the studied nanopolyamines are synthesized from spermine analogues. As the glypican-1 model agrees most with the known transport of other polycationic nanoparticles, future investigation into the role of glypican-1 in nanopolyamine transport is warranted. Additionally, as an alternative line of experimentation, caveolin-mediated endocytosis as a mechanism for nanopolyamine transport should be evaluated [8].

While the data from this study may indicate limited efficacy of nanopolyamines in polyamine transport-resistant cells, their utility as a combined polyamine metabolism and nucleic acid therapy remains. Their ability to induce polyamine catabolism and ROS damage are the molecular basis of their antitumor mechanism of action and fundamental to their activity. This will hopefully lead to renewed interest in moving forward, ultimately resulting in clinical trials for the new formulations of these novel polyamine analogues. Most importantly, this study provides evidence that endocytosis, possibly receptor-mediated endocytosis, is the likely mechanism responsible for the transport of higher order polyamines and their analogues. What now remains is the task of identifying the possible receptors and the other molecular players mediating polyamine transport.

## 5. Conclusions

Polyamine analogues are a promising therapeutic for modulating aberrant polyamine metabolism in cancerous cells. The nanopolyamines developed from the analogues PG-11047 and DSS-BEN provide a novel form of polyamine analogue metabolic modulation and the potential to simultaneously introduce a secondary therapy as drug delivery vesicles. This study found that nanopolyamines were incapable of accumulating in polyamine transport-deficient cells and subsequently were unable to modulate their polyamine metabolism. These data indicate that while much larger in size, the nanopolyamines utilize the same polyamine transport as the parental analogues. While this discovery may limit the efficacy of nanopolyamines in cancer cells that have gained analogue resistance through polyamine transport downregulation, this information provides new evidence that endocytosis is a likely mechanism for higher order polyamine and analogue transport in mammalian cells.

## Figures and Tables

**Table 1:** IC_50_ values following nanopolyamine and parent analogue treatment.

Cell Line	Drug	IC_50_
A549G	PG-11047 Nano11047 BENSpm DSS-BEN	7.9 μM 28.4 μg/mL 1.6 μM 19.9 μg/mL
A549R	PG-11047 Nano11047 BENSpm DSS-BEN	>10 μM >25 μg/mL >10 μM >25 μg/mL
H157G	PG-11047 Nano11047 BENSpm DSS-BEN	12.8 μM 13.9 μg/mL 2.9 μM 14.65 μg/mL
H157R	PG-11047 Nano11047 BENSpm DSS-BEN	>10 μM >25 μg/mL >10 μM >25 μg/mL

## Data Availability

Data supporting reported results are contained within this article.
